# Right colon angiodysplasia and lower limb chronic obstructive arteropathy: simultaneous endovascular treatment

**DOI:** 10.1186/1471-2318-11-S1-A42

**Published:** 2011-08-24

**Authors:** G Patrizi, M Fazi, L Fiengo, G Di Rocco, F Pelle, D Giannotti, L Venturini, F Frezzotti, R Giordano, A Redler, FM Salvatori

**Affiliations:** 1 Dipartimento di Scienze Chirurgiche, Policlinico Umberto I, Università di Roma“La Sapienza”, Rome, Italy; 2 Dipartimento di Scienze Chirurgiche, Policlinico Umberto I, Università di Roma“La Sapienza”, Rome, Italy

## Background

Intestinal angiodyspalsia is a vascular artero-venous malformation of the enteric wall smaller than 1 cm whose main symptom is enterorrhagy.

Diagnosis is based on colonoscopy and mesenteric selective arteriography. Recently, spiral angio-CT has reached a good diagnostic sensitivity and specificity.

Radical treatment is still surgical resection. Endoscopic approaches are frequently employed even though relapse of hemorrhage and perforation can occur. Super-selective embolization represents another possible therapeutic option, despite the low risk of segmentary intestinal ischemia.

## Materials and methods

We present the case of a 70 year old patient, with a past history of hypertension, myocardial infarction, who came to our attention for a chronic obstructive arteriopathy of the lower extremities and claudicatio at 100 m. The patient also displayed an hypochromic anemia (Hb=7g/dL); no history of evident bleeding could be demonstrated. Ultrasonography showed the left common iliac artery obstruction and patent femoral, popliteal and tibial arteries.

The patient underwent colonoscopy that revealed a reddish area in the caecum that could be referred to angiodysplasia, and two small peduncolated polyps (0.5 and 0.8 mm) that were resected. Histology gave evidence of a low-grade tubular adenoma. Abdominal and lower limbs arteriography confirmed the ultrasonographic suspicion and we therefore proceeded with primary stenting of the iliac obstruction. At the same time, selective arteriography of the superior mesenteric artery was carried out, showing an angiographic appearance compatible with angiodysplasia of the terminal branches of the ileo-colic artery. Super-selective catheterization and embolization of the vascular malformation with metallic coils was then attempted successfully.

## Results

Endoscopic and angiographic follow-up showed the disappearing of the angiodysplastic area. After 6 and 12 months, no evidence of relapse could be identified at endoscopy. An occult blood test in the stools was negative and complete regression of symptoms was steadily achieved.

## Conclusions

Super-selective embolization is a safe and effective alternative option to surgical resections and endoscopic approach for the treatment of colic angiodysplasia.

